# Spatial correlation of covid-19 with intensive care unit beds in Paraná

**DOI:** 10.11606/s1518-8787.2022056003868

**Published:** 2022-03-23

**Authors:** Eduardo Rocha Covre, Natan David Pereira, Natan Nascimento de Oliveira, Patrícia Bossolani Charlo, Magda Lúcia Félix de Oliveira, Rosana Rosseto de Oliveira, Lígia Carreira, Luiz Augusto Facchini, João Ricardo Nickenig Vissoci, Maria Aparecida Salci

**Affiliations:** I Universidade Estadual de Maringá Programa de Pós-Graduação em Enfermagem Maringá PR Brasil Universidade Estadual de Maringá. Programa de Pós-Graduação em Enfermagem. Maringá, PR, Brasil; II Centro Universitário Ingá Departamento de Enfermagem Maringá PR Brasil Centro Universitário Ingá. Departamento de Enfermagem. Maringá, PR, Brasil; III Universidade Federal de Pelotas Programa de Pós-Graduação em Epidemiologia e Saúde da Família Pelotas RS Brasil Universidade Federal de Pelotas. Programa de Pós-Graduação em Epidemiologia e Saúde da Família. Pelotas, RS, Brasil; IV Duke University Duke University Medical Center Durham NC United States Duke University. Duke University Medical Center. Durham, NC, United States

**Keywords:** COVID-19, complications, Intensive Care Units, supply & distribution, Spatial Analysis, Health Services Needs and Demand, Ecological Studies

## Abstract

**OBJECTIVE:**

To analyze the spatial correlation between confirmed cases of covid-19 and the intensive care unit beds exclusive to the disease in municipalities of Paraná.

**METHODS:**

This is an epidemiological study of ecological type which used data from the Epidemiological Report provided by the Department of Health of Paraná on the confirmed cases of covid-19 from March 12, 2020, to January 18, 2021. The number of intensive care beds exclusive to covid-19 in each municipality of Paraná was obtained by the *Cadastro Nacional de Estabelecimentos de Saúde* (CNES - National Registry of Health Establishments), provided online by the *Departamento de Informática do Sistema Único de Saúde* (Datasus - Informatics Department of the Brazilian Unified Health System). The Bivariate Moran’s Index (local and global) was used to analyze the intensive care bed variable and spatial correlation, with a 5% significance level. LISA Map was used to identify critical and transition areas.

**RESULTS:**

In the analyzed period, we found 499,777 confirmed cases of covid-19 and 1,029 intensive care beds exclusive to the disease in Paraná. We identified a positive spatial autocorrelation between the confirmed cases of covid-19 (0.404–p ≤ 0.001) and intensive care beds exclusive to the disease (0.085–p ≤ 0.001) and disparities between the regions of Paraná.

**CONCLUSION:**

Spatial analysis indicated that confirmed cases of covid-19 are related to the distribution of intensive care beds exclusive to the disease in Paraná, allowing us to find priority areas of care in the state regarding the dissemination and control of the disease.

## INTRODUCTION

Infectious diseases are still a major threat to global public health. In December 2019, China recorded the first cases of an infection caused by the new zoonotic coronavirus named coronavirus-2 (SARS-CoV-2)^1^. Infections caused by this type of coronavirus are recurrent, similarly to 2002 and 2012 epidemics of SARS-CoV and MERS-CoV, respectively, which infected more than 10,000 people worldwide^2,3^.

The new infection, first called 2019-nCov, is now called coronavirus disease 2019 (covid-19)^4^, considered a worldwide pandemic because of its high transmissibility and rapid evolution which overcomes continental barriers^1,5^. Estimates show that, as of April 26, 2021, more than 146.8 million people had been diagnosed with the disease worldwide, out of which 2.1% had died^6^.

Covid-19 mainly affects the human respiratory system, often evolving to acute respiratory distress and diffuse alveolar damage. Those infected have a high risk of mortality and therefore require intensive care^1,7^. A retrospective study conducted in China indicated that about 25% of infected people developed severe covid-19, out of which 80% required hospitalization in intensive care units (ICU)^8^. Many ICU beds are therefore essential to attend all those infected with severe covid-19.

Brazil was the first Latin-American country to report a case of covid-19, on February 26, 2020^9^. As of April 26, 2021, Brazil had more than 390,000 confirmed deaths and 14.3 million confirmed cases of the disease, ranking second in the world ranking of confirmed cases of contamination by the new coronavirus^6^. The state of Paraná had reported more than 21,000 deaths and 925,000 confirmed cases of covid-19^10^.

However, cases of covid-19 are distributed unevenly among regions and municipalities. The first cases were identified in national capitals and, over time, new cases appeared in more distant locations because of the virus’ community transmission^8^.

To understand covid-19 propagation, we must learn how it distributes itself spatially, from large urban centers to more remote and less developed areas of the country. Furthermore, observing how covid-19 expands geographically together with exclusive ICU beds can help understand accessibility to intensive care, essential for severe patients.

Thus, the following research question was asked: “Does the distribution of ICU beds exclusive to covid-19 reflect the spatial distribution of cases of the disease in municipalities of Paraná?”. This study therefore sought to analyze the spatial correlation between confirmed cases of covid-19 and intensive care unit beds exclusive to the disease in the municipalities of Paraná.

## METHODS

This is an epidemiological study of ecological type on the prevalence of confirmed cases of covid-19 and ICU beds exclusive to the disease in the municipalities of the state of Paraná. Confirmed cases of the disease, recorded between March 12, 2020 (first cases registered in the state) and January 18, 2021 (date of data collection for the study), were considered according to place of residence.

**Figure 1:**
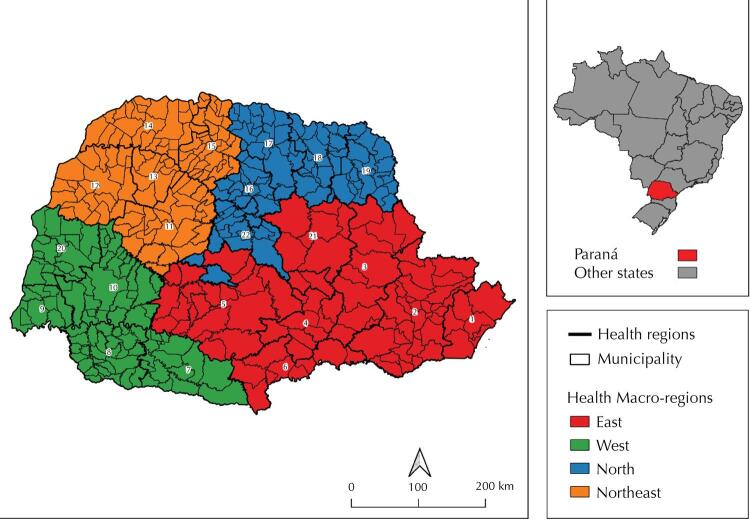
Distribution of macro-regions and health regions in Paraná. Paraná, Brazil, 2021.

Source: survey data, 2021.

Paraná is one of the three states in Southern Brazil, with an estimated population of 11,516,840 inhabitants in 2020^11^. Its extensive border region meets the states of Santa Catarina, São Paulo, and Mato Grosso do Sul, the countries Argentina and Paraguay, and the Atlantic Ocean. The state is organized in 399 municipalities grouped into 22 health regions (HR) and four health macro-regions (Figure 1), all integrated in the logic of health care network^12^.

Data on the number of confirmed cases of the disease and population data were obtained from the Epidemiological Report provided by the *Secretaria de Estado da Saúde do Paraná* (SESA — Department of Health of Paraná) on January 18, 2021^13^. The number of ICU beds exclusive to covid-19 in each municipality of Paraná was obtained by the *Cadastro Nacional de Estabelecimentos de Saúde* (CNES – National Registry of Health Establishments) — physical resources, provided online by the *Departamento de Informática do Sistema Único de Saúde* (Datasus — Informatics Department of the Brazilian Unified Health System). Data were collected on January 18, 2021, at 8 p.m., considering adult and pediatric ICU beds of public and private hospitals. Data are constantly updated and the ICU beds exclusive to covid-19 available until December 2020 were considered^14^.

Municipalities of the state of Paraná were considered as the units of analysis for spatial distribution and autocorrelation analyses. The cartographic basis with the limits of the municipalities was obtained from the website of the Brazilian Institute of Geography and Statistics (IBGE). Considering that the number of cases and beds could change randomly, prevalence was calculated by the smoothed ratio of the number of cases and the number of ICU beds according to the estimated population of each municipality, multiplied by 100 and 100,000, respectively. Rate smoothing was conducted by queen contiguity-based spatial weights, which determine neighborhood from any common border to the municipalities.

The results were presented in choropleth maps, organized by the Jenks natural breaks optimization method. This type of classification ensures data homogeneity and heterogeneity by “breaking” the categories according to the variance of different groups, minimizing the variance within the groups themselves^15^.

The statistical analysis of spatial dependence used Moran I’s spatial autocorrelation coefficient, which is subdivided into Global Moran Index and Local Moran Index.

Firstly, the Global Moran Index was calculated. Statistics proposed by Moran^16^ were used to analyze the spatial distribution pattern of the variable according to municipality. This index varies between -1 and 1, in which values close to zero indicate no spatial autocorrelation, positive values indicate positive spatial autocorrelation, and negative values indicate negative autocorrelation. Then, a pseudo p-value was estimated for the Global Moral index from 999 permutations.

The Local Moran Index was used to identify clusters of areas with similar risks for the occurrence of the outcome of interest if the Global Moran Index identified a significant spatial autocorrelation. This index allows analyzing the extent to which the value of a variable for a given area is similar or different to its neighboring areas^16^.

Clusters formed from the analysis of the Local Moran Index can be divided into four parameters: high-high, which represents high-rate municipalities and neighbors; low-low, of low-rate municipalities and neighbors; low-high, of low-rate cities and high-rate neighbors; and high-low, of high-rate cities and low-rate neighbors. These data were presented by the LISA (Local Indicators of Spatial Association) Map, which allows expressing local spatial dependence patterns using choropleth maps^17^.

The spatial correlation analysis between confirmed cases of covid-19 and ICU beds exclusive to the disease was conducted by Bivariate Moran’s Index. This index designates whether two variables in a given location in space are associated with each other^17^. The significance level was considered as 5%. Statistical analyses were performed by the GeoDa software version 1.18 and the maps were elaborated in the QGIS software version 3.10.

This study was not submitted to the *Comissão Nacional de Ética em Pesquisa* (Conep — National Research Ethics Committee) according to resolution no. 510, of April 7, 2016, since it uses data obtained from secondary sources, does not identify research subjects, and is available in public domain.

## RESULTS

According to the epidemiological report of the Department of Health of Paraná, until January 18, 2021, 499,777 cases of covid-19 had been confirmed in the state, with a prevalence of 4.34 cases per 100 inhabitants. Health regions (HR) of Foz do Iguaçu (9th HR) and União da Vitória (6th HR) had the highest and lowest prevalence of cases, with 7.58 and 1.84 cases per 100 inhabitants, respectively (Table).

**Table:** Prevalence of confirmed cases of covid-19a and ICU beds exclusive to the disease in health regions. Paraná, Brazil, March 2020 to January 2021.

Health Region (HR)	Population	Confirmed cases	ICU beds^b^	Prevalence of cases^c^	Beds per inhabitants^d^
1^st^ HR Paranaguá	299,824	16,903	20	5.64	6.68
2^nd^ HR Metropolitana	3,693,891	157,945	416	4.27	11.26
3^rd^ HR Ponta Grossa	642,624	24,764	30	3.85	4.67
4^th^ HR Irati	176,074	5,558	4	3.15	2.27
5^th^ HR Guarapuava	457,280	12,331	40	2.70	8.75
6^th^ HR União da Vitória	178,277	3,285	6	1.84	3.37
7^th^ HR Pato Branco	268,563	11,550	28	4.30	10.43
8^th^ HR Francisco Beltrão	359,601	18,014	10	5.00	2.78
9^th^ HR Foz do Iguaçu	405,246	30,737	50	7.58	12.34
10^th^ HR Cascavel	554,233	27,861	46	5.03	8.30
11^th^ HR Campo Mourão	327,595	10,404	31	3.17	9.46
12^th^ HR Umuarama	277,003	12,076	30	4.36	10.83
13^th^ HR Cianorte	162,273	6,502	8	4.00	4.93
14^th^ HR Paranavaí	277,060	7,515	10	2.71	3.61
15^th^ HR Maringá	847,559	42,190	94	4.98	11.09
16^th^ HR Apucarana	387,414	13,469	50	3.48	12.91
17^th^ HR Londrina	972,283	47,998	80	4.94	8.23
18^th^ HR Cornélio Procópio	221,744	7,430	8	3.35	3.61
19^th^ HR Jacarezinho	289,587	7,772	10	2.68	3.45
20^th^ HR Toledo	401,772	22,640	30	5.63	7.47
21^st^ HR Telêmaco Borba	189,750	9,516	24	5.01	12.65
22^nd^ HR Ivaiporã	127,237	3,317	4	2.60	3.14
**Paraná**	**11,516,840**	**499,777**	**1,029**	**4.34**	**8.93**

Source: Preparation based on data from the *Secretaria de Estado da Saúde do Paraná* (SESA — Department of Health of Paraná) and the Brazilian Ministry of Health’s *Departamento de Informática do Sistema Único de Saúde* (Datasus — Informatics Department of the Brazilian Unified Health System).

^a^ Coronavirus disease 2019.

^b^ ICU: intensive care unit, exclusive to covid-19.

^c^ Per 100 inhabitants.

^d^ Per 100,000 inhabitants.

Paraná had 1,029 ICU beds exclusive to covid-19, with a prevalence of 8.93 beds per 100,000 inhabitants. Apucarana (16^th^ HR) and Irati (4^th^ HR) recorded the highest and lowest prevalence of beds, with 12.91 and 2.27 beds per 100,000 inhabitants, respectively (Table).

**Figure 2:**
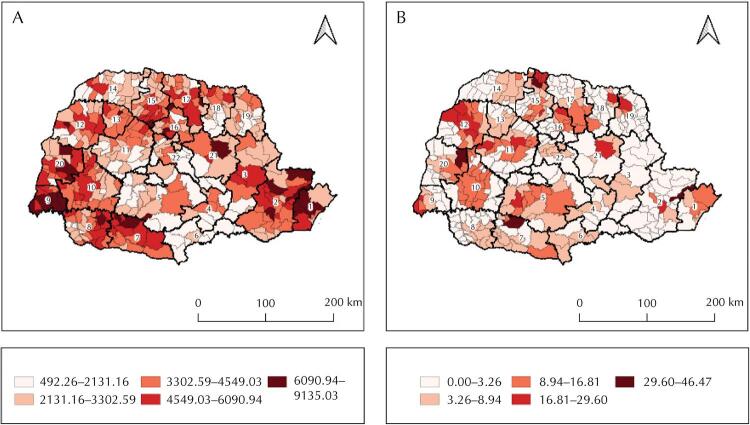
Spatial distribution of the smoothed prevalence of confirmed cases of covid-19 (A) and intensive care unit beds (B) in municipalities and health regions. Paraná, Brazil, March 2020 to January 2021.

Confirmed cases of covid-19 and ICU beds exclusive to the disease were unevenly distributed in Paraná. Rates of covid-19 cases were lower in the 5^th^ (Guarapuava) and the 11^th^ (Campo Mourão) health regions and prevalent in the 1^st^ (Paranaguá) and the 9^th^ (Foz do Iguaçu). Of the 399 municipalities of Paraná, few had a high prevalence of ICU beds exclusive to covid-19 whereas 367 had none (Figure 2).

**Figure 3:**
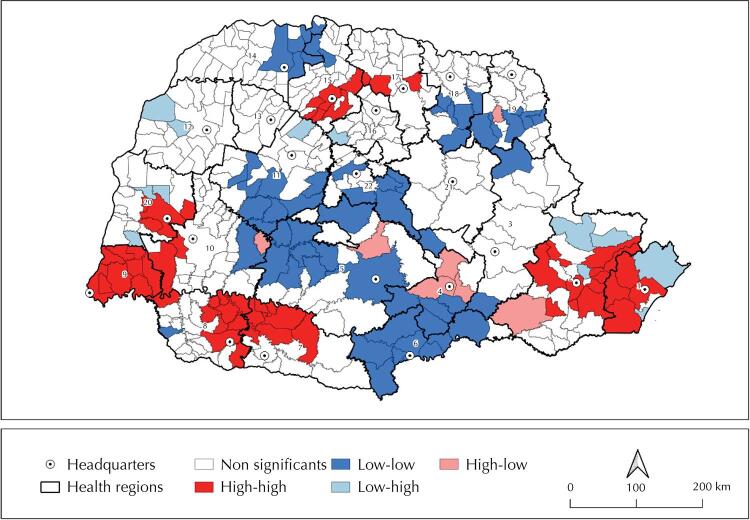
Spatial autocorrelation of confirmed cases of covid-19 in headquarter municipalities of health regions and neighboring regions, according to the analysis of Univariate Local Moran’s I (LISA — local indicator of spatial association). Paraná, Brazil, March 2020 to January 2021.

The Global Moran Index indicated a positive spatial autocorrelation of 0.404 (p ≤ 0.001) for covid-19 cases in Paraná. Municipalities of significant covid-19 rates and place of residence formed clusters. In total, 265 municipalities were not significant, 55 were of high-high pattern, 60 low-low, 12 low-high, and seven high-low (Figure 3).

We identified three high-high clusters, one of which included the entire 9^th^ HR (Foz do Iguaçu), with conglomerate municipalities from the 10^th^ HR (Cascavel) and 20^th^ HR (Toledo); another was formed by cities of the 1^st^ HR (Paranaguá) and 2^nd^ HR (Curitiba); and the other by municipalities of the 7^th^ HR (Pato Branco) and 8^th^ HR (Francisco Beltrão). Northern Paraná has a fourth high-high cluster, representing the regional cities of Maringá (15^th^HR) and Londrina (17^th^ HR) (Figure 3).

A large low-low cluster was formed with almost all municipalities of the 6^th^ HR (União da Vitória) and some municipalities in the 3^rd^ (Ponta Grossa), 4^th^ (Irati), 5^th^ (Guarapuava), 11^th^ (Campo Mourão), and 12^th^ (Umuarama) HRs, extended along South-Central Paraná. Moreover, Northern Paraná had two other low-low clusters, one of them formed by 14 municipalities in the 18^th^ (Cornélio Procópio) and 19^th^ (Jacarezinho) HRs and another composed by six cities of the 14^th^ (Paranavaí) HR and a city of the 15^th^ (Maringá) HR. Few municipalities along the state had low-high and high-low values (Figure 3).

In the bivariate analysis, confirmed cases of covid-19 collected from the place of residence of those infected were positively or directly correlated with the smoothed distribution of ICU beds, with index 0.085 (p ≤ 0.001). Despite the statistical significance, the number of ICU beds and confirmed cases of covid-19 were little correlated, following the pattern of most municipalities in Paraná. Of all municipalities (399), 265 (66.4%) showed no significant correlation between number of beds and covid-19 prevalence. The remaining 134 (33.6%) municipalities formed clusters, two of which stand out: one is formed by municipalities with a strong correlation between high number of beds and high prevalence of covid-19 whereas another is formed by municipalities with low number of beds and low prevalence of covid-19. Correlation was 1.105 (p < 0.05) in the high-high cluster and 0.499 (p < 0.005) in the low-low cluster.

**Figure 4:**
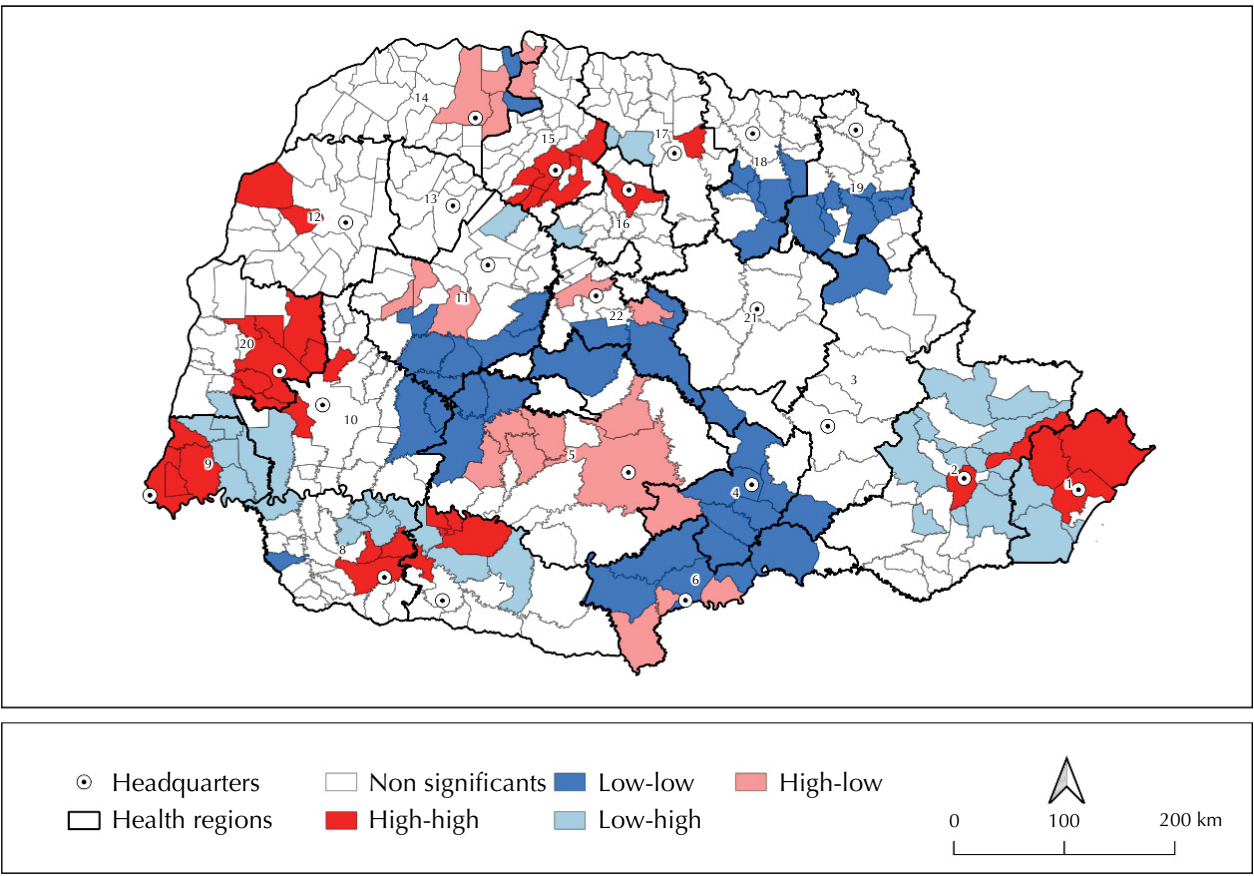
Spatial distribution of the correlation between the rate of intensive care unit beds exclusive to covid-19 and confirmed cases of the disease, according to analysis of the Bivariate Local Moran’s I (LISA — local indicator of spatial association). Paraná, Brazil, March 2020 to January 2021.

Among clusters of municipalities with a high number of ICU beds and a high prevalence of cases, Curitiba and Paranaguá stand out in the Eastern macro-region; Foz do Iguaçu and Toledo in the Western macro-region; Umuarama and Maringá in the Northwestern macro-region; and Londrina and Apucarana in the Northern macro-region (Figure 4). Figure 4 shows the cluster of municipalities with a low number of ICU beds and low prevalence of covid-19 cases, particularly Cornelius Procópio, Jacarezinho, and Ivaiporã in the Northern macro-region; Irati, Guarapuava, and União da Vitória in the Eastern macro-region; and Campo Mourão in the Northwestern macro-region. The Western macro-region shows a low-low cluster of municipalities Guaraniaçu and Diamante do Sul from the Cascavel region, which is however added to the Guarapuava regional pattern.

A group of municipalities with a high number of ICU beds and low prevalence of covid-19 cases formed outliers, including the municipalities of Colorado and Paranavaí and the high-low cluster of Rancho Alegre D’Oeste, Janiópolis, and Mamborê, in the Northwestern macro-region. In the Eastern macro-regional, Guarapuava, Turvo, Marquinho, Laranjeiras do Sul, Cantagalo, and Goioxim also formed a high-low cluster. Geographically, high-low clusters are on the periphery of low-low clusters whereas low-high clusters surround high-high clusters (Figure 4).

## DISCUSSION

We found no studies in the scientific literature which correlate confirmed cases of covid-19 with intensive care unit beds in the state of Paraná. This correlation, analyzed by spatial analysis tools, allowed identifying vulnerable regions and municipalities in the state, understanding the relationship between the event and neighboring municipalities, and analyzing the covid-19 behavior in Paraná according to the availability of exclusive ICU beds.

A similar study analyzed the distribution of covid-19 cases and exclusive care beds in the state of Ceará^18^, showing that the disease went beyond the capital city Fortaleza and its metropolitan region and that regions with high rates of contamination lacked beds.

The number of confirmed cases and deaths by covid-19 worldwide increases constantly and Brazil is one of the disease’s epicenters. Such growth is caused by the virus’ high transmissibility and dissemination and the populations’ lack of prior immunity and vaccination against it^20^.

Until January 18, 2021, the prevalence of confirmed cases in Brazil was 4,039 per 100,000 inhabitants. Covid-19 cases increased in all regions of the country. The South had a rate of 5,470 confirmed cases per 100,000 inhabitants, of which Paraná had the lowest rate (4,339 confirmed cases – according to the results of this study), followed by Rio Grande do Sul (4,717) and Santa Catarina (7,915)^21^. Southern states have had the same ranking on the number of covid-19 cases since the beginning of the pandemic, according to results from other studies^21^.

New cases of covid-19 are growing in the South and particularly in the state of Paraná. Though the number of confirmed cases in this region is less worrisome than in other regions of the country^13^, specific actions must be developed to collectively control the disease and to contain the spread of the virus between municipalities and health regions.

Our results indicate that confirmed cases of covid-19 were unequally distributed among municipalities and health regions of Paraná because of the date in which the disease first occurred, demographic and population density, age distribution and population characteristics, health service conditions, control of disease dissemination in communities, practice and reliability of notification of cases, and diagnostic capacity^24^.

In Paraná, municipalities of the Foz do Iguaçu (9^th^ HR), Paranaguá (1^st^ HR), and Francisco Beltrão (8^th^ HR) health regions had the highest prevalence of confirmed cases of covid-19, above the state average.

Cases of covid-19 from the metropolitan region of Curitiba, located in the Eastern macro-region, directly affect Paranaguá (neighboring city of the capital and border of the state of São Paulo)^21^. Cases were internalized in the 9^th^ and 8^th^ HRs of the Western macro-region since these regions are considerable commercial centers of the state, linked to the capital city by BR-376, BR-476, BR-153, and BR-277^21^. Therefore, to understand the territorial expansion of covid-19, we must learn about social, economic, and commercial networks and dependencies and the travel and transportation flow within the boundaries of a region or state^25^.

The Eastern macro-region of Paraná, specifically the municipalities of the 6^th^ HR, União da Vitória, had the lowest prevalence of confirmed cases of covid-19 (1,843 confirmed cases per 100,000 inhabitants – lower than the state average). This region also had significant local spatial dependence, similarly to neighboring regions.

However, these regions still require more actions to prevent and control new cases caused by the commuting of infected people and consequent transmission of the virus. The population should be encouraged to maintain hygiene, distancing, and social isolation measures since the virus spreads rapidly with community transmission^8^. Moreover, the number of infected people has increased greatly in municipalities and regions that first had a low prevalence of cases^29^.

Brazil has a rate of 9.77 ICU beds exclusive to covid-19 per 100,000 inhabitants. The Southeast has the highest rate in the country (10.90 beds) whereas the South has a prevalence of 10.14. Among Southern states, Santa Catarina has the highest rate (12.82), followed by Rio Grande do Sul (9.59) and Paraná (8.99)^14^. We thus found that Paraná is the Southern state with the lowest rate of ICU beds exclusive to covid-19, corroborating with a previous study^28^.

This study’s results showed that municipalities in the 7^th^ HR — Pato Branco (52.17), 2^nd^ HR — Curitiba (45.78), and 20^th^ HR — Toledo (41.99) had a higher prevalence of exclusive ICU beds per 100,000 inhabitants. Similarly to the prevalence of confirmed covid-19 cases, the distribution of exclusive beds differed among municipalities and health regions of Paraná, considering that Curitiba, Guarapuava, Pato Branco, Campo Mourão, Maringá, and Toledo had more beds exclusive to covid-19.

Bivariate analysis identified a cluster in which the high prevalence of covid-19 cases correlates positively with the high availability of ICU beds exclusive to the disease. Findings indicate that large and medium-sized municipalities with higher demographic density and high rates of the disease are often the headquarters of health regions (following the principle of regionalization of the Brazilian Unified Health System — SUS). Their distribution and centralization of ICU beds can be considered as valid strategies for a homogeneous and equal distribution. However, since the disease evolves rapidly, patients should have fast access to health services^24,30,31^. New studies on the spatial dimensioning of care services for severe cases of covid-19 could greatly contribute to the knowledge about this phenomenon.

This study has limitations. Data was collected in a single period, but the number of confirmed cases is constantly updating and therefore increasing. Regarding the number of beds exclusive to covid-19, we considered only the beds available in Paraná until the time of data collection. We identified no other studies on the geographic distribution of covid-19 in Paraná that could be used to expand and compare with the findings of this study. However, our study could support future epidemiological research on the spatial analysis of covid-19 and help state and municipal health managers in decision-making.

Nevertheless, our results allowed identifying the direct spatial autocorrelation of covid-19 cases in the state of Paraná and their positive correlation with ICU beds exclusive to the disease, indicated by the uneven distribution of cases and beds between municipalities and health regions. Finally, the Local Moran Index identified clusters which emphasize priority areas of care in the state.
