# Supplementary material for: Spatial correlation of covid-19 with intensive care unit beds in Paraná
Source: Rev Saude Publica. 2022 Mar 23;56:14. doi: 10.11606/s1518-8787.2022056003868 (PMC8963746; doi:10.11606/s1518-8787.2022056003868)
Supplement: Translation

# Translation: Correlação espacial da covid-19 com leitos de unidades de terapia intensiva no Paraná

**Authors:** Eduardo Rocha Covre, Natan David Pereira, Natan Nascimento de Oliveira, Patrícia Bossolani Charlo, Magda Lúcia Félix de Oliveira, Rosana Rosseto de Oliveira, Lígia Carreira, Luiz Augusto Facchini, João Ricardo Nickenig Vissoci, Maria Aparecida Salci

**Affiliations:** I Brasil Universidade Estadual de Maringá. Programa de Pós-Graduação em Enfermagem. Maringá, PR, Brasil; II Brasil Centro Universitário Ingá. Departamento de Enfermagem. Maringá, PR, Brasil; III Brasil Universidade Federal de Pelotas. Programa de Pós-Graduação em Epidemiologia e Saúde da Família. Pelotas, RS, Brasil; IV United States Duke University. Duke University Medical Center. Durham, NC, United States

**Keywords:** COVID-19, complicações, Unidades de Terapia Intensiva, provisão & distribuição, Análise Espacial, Necessidades e Demandas de Serviços de Saúde, Estudos Ecológicos

## Abstract

**OBJETIVO:**

Analisar a correlação espacial entre os casos confirmados de covid-19 com os leitos de unidades de terapia intensiva exclusivos para a doença nos municípios do Paraná.

**MÉTODO:**

Trata-se de um estudo epidemiológico, do tipo ecológico que utilizou dados do Informe Epidemiológico fornecido pela Secretaria de Estado da Saúde do Paraná sobre os casos confirmados de covid-19, no período de 12 de março de 2020 a 18 de janeiro de 2021. A quantidade de leitos de terapia intensiva exclusivos para covid-19 de cada município paranaense foi obtida pelo Cadastro Nacional de Estabelecimentos de Saúde disponibilizado online pelo Departamento de Informática do Sistema Único de Saúde. A variável leito de terapia intensiva foi analisada pelo Índice Bivariado de Moran (local e global). Para a identificação de áreas críticas e de transição utilizou-se o LISA Map. Para avaliar a correlação espacial foi utilizado o Índice Bivariado de Moran, considerando o nível de significância de 5%.

**RESULTADOS:**

No período analisado foram confirmados 499.777 casos de covid-19 no Paraná e identificados 1.029 leitos de terapia intensiva exclusivos para a doença entre os municípios do estado. Foi identificado autocorrelação espacial positiva entre os casos confirmados de covid-19 (0,404–p ≤ 0,001) com os leitos de terapia intensiva exclusivos para a doença (0,085–p ≤ 0,001) e disparidades entre as regiões do Paraná.

**CONCLUSÃO:**

A análise espacial permitiu confirmar a relação entre os casos confirmados de covid-19 e a distribuição de leitos de terapia intensiva exclusivos para a doença no Paraná e possibilitou identificar áreas prioritárias de atenção no estado, relacionadas à disseminação e controle da doença.

## INTRODUÇÃO

As doenças infecciosas ainda representam uma grande ameaça à saúde pública mundial. Na China, em dezembro de 2019, foram registrados os primeiros casos de uma infecção causada pelo novo coronavírus zoonótico nomeado de síndrome respiratória aguda grave coronavírus-2 (SARS-CoV-2)^1^. Infecções causadas por esse tipo de coronavírus são recorrentes, como nas epidemias ocorridas em 2002 e 2012 por SARS-CoV e MERS-CoV, respectivamente, responsáveis pela infecção de mais de 10 mil pessoas em todo o mundo^2,3^.

A nova infecção, de início chamada 2019-nCov é denominada hoje por doença de coronavírus 2019 (covid-19)^4^ e, devido ao alto poder de transmissibilidade e evolução rápida, tendo ultrapassado barreiras continentais, é considerada uma pandemia mundial^1,5^. Estimativas pontuam que no mundo, até 26 de abril de 2021, mais de 146,8 milhões de pessoas foram diagnosticadas com a doença, sendo que aproximadamente 2,1% delas evoluíram a óbito^6^.

A covid-19 tem como alvo principal o sistema respiratório humano, o que pode evoluir em sua maioria para desconforto respiratório agudo e dano alveolar difuso, com grande potencial de morte aos infectados, necessitando-se de cuidados intensivos^1,7^. Evidências trazidas por um estudo retrospectivo realizado na China demonstraram que cerca de 25% das pessoas diagnosticadas com covid-19 evoluíram para a forma grave da doença e 80% delas necessitaram de internamento em leitos de unidade de terapia intensiva (UTI)^8^. Diante disso, nota-se a necessidade de uma quantidade expressiva de leitos de UTI para atender à demanda da população que desenvolve a forma grave da patologia.

O Brasil foi o primeiro país da América Latina a notificar a covid-19, no dia 26 de fevereiro de 2020^9^. Até 26 de abril de 2021 foram confirmados mais de 390 mil óbitos e 14,3 milhões de casos confirmados da doença, colocando o país na segunda posição do ranking mundial de número de casos confirmados de contaminação pelo novo coronavírus^6^. No estado do Paraná, registram-se mais de 21 mil óbitos e 925 mil casos confirmados de covid-19^10^.

Entretanto, a distribuição de casos da covid-19 não se dá de forma homogênea nas regiões e nos municípios. Os primeiros casos foram identificados nas capitais dos países e, com o passar do tempo, novos casos começaram a ser detectados em regiões e municípios mais longínquos, por consequência da transmissão comunitária do vírus^8^.

Conhecer como a doença se distribui espacialmente em uma região permite a compreensão da sua propagação, no caso da covid-19, a dinâmica dos grandes centros urbanos para áreas mais remotas e menos desenvolvidas do interior. Ainda, observar como a doença se dispersa geograficamente aliado à expansão de leitos de UTI exclusivos para covid-19 facilita o entendimento sobre a acessibilidade a esse tipo de atendimento, que pode ser crucial ao paciente grave.

Diante desse cenário surgiu a seguinte questão de pesquisa: A distribuição de leitos de UTI exclusivos para covid-19 reflete a distribuição espacial de casos da doença nos municípios do Paraná? Assim, este estudo teve como objetivo analisar a correlação espacial entre os casos confirmados de covid-19 com os leitos de unidades de terapia intensiva exclusivos para a doença nos municípios do Paraná.

## MÉTODO

Trata-se de um estudo epidemiológico, do tipo ecológico, sobre a prevalência de casos confirmados de covid-19 e de leitos de UTI exclusivos para a doença nos municípios do estado do Paraná. Foram considerados os casos confirmados da doença, por local de residência, registrados entre 12 de março de 2020 (primeiros casos registrados no estado) e 18 de janeiro de 2021 (data da coleta dos dados para o estudo).

O Paraná é um dos três estados da região sul do Brasil, com população estimada em 11.516.840 habitantes no ano de 2020^11^. Possui extensa região de fronteira com os estados de Santa Catarina, São Paulo e Mato Grosso do Sul, e com os países Argentina, Paraguai e o Oceano Atlântico. Está organizado em 399 municípios, agrupados em 22 regionais de saúde (RS), articuladas em quatro macrorregionais de saúde (Figura 1), todas integradas na lógica de rede de atenção à saúde^12^.

**Figura 1:** Distribuição das macrorregionais e das regionais de saúde do Paraná. Paraná, Brasil, 2021.

Fonte: dados da pesquisa, 2021.

Os dados sobre o número de casos confirmados da doença e dados populacionais foram obtidos por meio do Informe Epidemiológico disponibilizado pela Secretaria de Estado da Saúde do Paraná (SESA) no dia 18 de janeiro de 2021^13^. O número de leitos de UTI exclusivos para covid-19 foi obtido do cadastro nacional de estabelecimentos de saúde (CNES – recursos físicos) disponibilizado online pelo departamento de informática do sistema único de saúde (Datasus). A coleta dos dados foi realizada no dia 18 de janeiro de 2021, às 20 horas, e foram considerados os leitos de UTI adulta e pediátrica de hospitais públicos e privados. Ressalta-se que os dados são atualizados constantemente e foram considerados os leitos de UTI exclusivos para covid-19 disponíveis até dezembro de 2020^14^.

Para a análise de distribuição e autocorrelação espacial, os municípios do estado do Paraná foram considerados como as unidades de análise. A base cartográfica com os limites dos municípios foi obtida no site do Instituto Brasileiro de Geografia e Estatística (IBGE). Considerando a possibilidade de flutuações aleatórias do número de casos e leitos, o cálculo da prevalência se deu pela razão suavizada do número de casos da doença e do número de leitos de UTI, pela população estimada de cada município, multiplicado por 100 e 100 mil, respectivamente. A suavização da taxa se deu pelo método de peso de contiguidade do tipo rainha, que determina vizinhança a partir de qualquer fronteira comum aos municípios.

Os resultados foram apresentados em mapas coropléticos, organizados a partir das quebras naturais de Jenks. Esse tipo de classificação garante a homogeneidade e a heterogeneidade dos dados, ao realizar a “quebra” das categorias de acordo com a variância de diferentes grupos, minimizando a variância dentro dos próprios grupos^15^.

Para a análise estatística da dependência espacial foi utilizado o Coeficiente de Autocorrelação Espacial de Moran, que é subdividido nos Índices de Moran Global e Índice de Moran Local.

Primeiramente foi calculado o Índice de Moran Global. A estatística proposta por Moran^16^ foi utilizada para analisar o padrão da distribuição espacial da variável segundo o município. O valor desse índice varia entre -1 e 1, sendo que valores próximos de zero, indicam a ausência de autocorrelação espacial, enquanto valores positivos indicam autocorrelação espacial positiva e valores negativos indicam autocorrelação negativa. Além disso, foi estimado um pseudo p-valor para o Índice de Moral Global, a partir de 999 permutações.

Já o Índice de Moran Local foi utilizado para identificar aglomerados de áreas com riscos semelhantes para ocorrência do desfecho de interesse, caso fosse identificada uma autocorrelação espacial significativa pelo Índice de Moran Global. Tal índice permite analisar até que ponto o valor de uma variável para uma determinada área é similar ou diferente às suas áreas vizinhas^16^.

Os aglomerados formados a partir da análise do Índice de Moran Local podem ser divididos em quatro parâmetros: alto-alto, que representa municípios com altas taxas e vizinhos com altas taxas; baixo-baixo, que representa municípios com baixas taxas e vizinhos com taxas igualmente baixas; baixo-alto, representando cidades com baixas taxas e vizinhos com altas taxas; e alto-baixo, que identifica cidades que apresentam altas taxas e vizinhos com taxas baixas. Para apresentação desses dados foi utilizado o LISA (*Local Indice of Spatial Association*) *Map* que permite expressar os padrões de dependência espacial em nível local por meio de mapas cloropléticos^17^.

A análise de correlação espacial entre os casos confirmados de covid-19 e os leitos de UTI exclusivos para a doença foi realizada a partir do Índice Bivariado de Moran. Esse índice designa se há associação entre duas variáveis em um determinado local no espaço^17^. Foi considerado o nível de significância de 5%. As análises estatísticas foram realizadas pelo software GeoDa versão 1.18, e os mapas foram elaborados no software QGIS versão 3.10.

Por se tratar de um estudo com utilização de dados obtidos de fontes secundárias, sem identificação de sujeitos da pesquisa e cujo acesso é de domínio público, não foi necessária a submissão à Comissão Nacional de Ética em Pesquisa (Conep), conforme a resolução nº 510, de 7 de abril de 2016.

## RESULTADOS

De acordo com as informações do boletim epidemiológico da Secretaria de Saúde do Estado do Paraná, até o dia 18 de janeiro de 2021, foram confirmados 499.777 casos da doença no estado, com uma prevalência de 4,34 casos por 100 habitantes. As regionais de saúde (RS) de Foz do Iguaçu (9ª RS) e União da Vitória (6ª RS) apresentaram a maior e menor prevalência, registrando 7,58 e 1,84 casos por 100 habitantes, respectivamente (Tabela).

**Tabela:** Prevalência de casos confirmados de covid-19a e de leitos de UTI exclusivos para a doença nas regionais de saúde. Paraná, Brasil, março de 2020 a janeiro de 2021.

Regional de Saúde (RS)	População	Casos confirmados	Leitos de UTI^b^	Prevalência de casos^c^	Leitos por habitantes^d^
1ª RS Paranaguá	299.824	16.903	20	5,64	6,68
2ª RS Metropolitana	3.693.891	157.945	416	4,27	11,26
3ª RS Ponta Grossa	642.624	24.764	30	3,85	4,67
4ª RS Irati	176.074	5.558	4	3,15	2,27
5ª RS Guarapuava	457.280	12.331	40	2,70	8,75
6ª RS União da Vitória	178.277	3.285	6	1,84	3,37
7ª RS Pato Branco	268.563	11.550	28	4,30	10,43
8ª RS Francisco Beltrão	359.601	18.014	10	5,00	2,78
9ª RS Foz do Iguaçu	405.246	30.737	50	7,58	12,34
10ª RS Cascavel	554.233	27.861	46	5,03	8,30
11ª RS Campo Mourão	327.595	10.404	31	3,17	9,46
12ª RS Umuarama	277.003	12.076	30	4,36	10,83
13ª RS Cianorte	162.273	6.502	8	4,00	4,93
14ª RS Paranavaí	277.060	7.515	10	2,71	3,61
15ª RS Maringá	847.559	42.190	94	4,98	11,09
16ª RS Apucarana	387.414	13.469	50	3,48	12,91
17ª RS Londrina	972.283	47.998	80	4,94	8,23
18ª RS Cornélio Procópio	221.744	7.430	8	3,35	3,61
19ª RS Jacarezinho	289.587	7.772	10	2,68	3,45
20ª RS Toledo	401.772	22.640	30	5,63	7,47
21ª RS Telêmaco Borba	189.750	9.516	24	5,01	12,65
22ª RS Ivaiporã	127.237	3.317	4	2,60	3,14
**Paraná**	**11.516.840**	**499.777**	**1.029**	**4,34**	**8,93**

Fonte: Elaboração a partir dos dados da Secretaria de Estado da Saúde do Paraná (SESA) e Ministério da Saúde (departamento de informática do sistema único de saúde – Datasus).

^a^ Doença de coronavírus 2019.

^b^ UTI: unidade de terapia intensiva, exclusiva para covid-19.

^c^ Por 100 habitantes.

^d^ Por 100 mil habitantes.

Os leitos de UTI exclusivos para covid-19 somaram 1.029 no estado, com prevalência de 8,93 leitos por 100 mil habitantes. Nas regionais de saúde de Apucarana (16ª RS) e Irati (4ª RS) registraram a maior e menor prevalência de leitos, apresentando 12,91 e 2,27 leitos por 100 mil habitantes, respectivamente (Tabela).

Foram encontradas disparidades no estado na distribuição de casos confirmados da covid-19 e leitos de UTI exclusivos para a doença. Regionais de saúde como a 5ª (Guarapuava) e a 11ª (Campo Mourão) apresentaram menores taxas de casos, enquanto a 1ª (Paranaguá) e a 9ª (Foz do Iguaçu) obtiveram alta prevalência dos mesmos. Com relação aos leitos, poucos municípios, espalhados pelo estado, apresentaram uma alta prevalência. Dos 399 municípios do Paraná, 367 deles não apresentou nenhum leito de UTI exclusivo para covid-19 (Figura 2).

**Figura 2:** Distribuição espacial da prevalência suavizada de casos confirmados da covid-19 (A) e de leitos de unidades de terapia intensiva (B) nos municípios e regionais de saúde. Paraná, Brasil, março de 2020 a janeiro de 2021.

O Índice de Moran Global apontou autocorrelação espacial positiva para os casos de covid-19 no estado, com valor de 0,404 (p ≤ 0,001). *Clusters* foram formados a partir dos aglomerados constituídos pelos municípios que apresentaram significância quanto à relação das taxas de covid-19 e o local de residência. No total, 265 municípios não demonstraram significância, 55 apresentaram padrão alto-alto, 60 baixo-baixo, 12 baixo-alto e sete alto-baixo (Figura 3).

**Figura 3:** Autocorrelação espacial dos casos confirmados de covid-19 em municípios sede de regionais de saúde e vizinhos, segundo a análise do Índice Local de Moran (LISA) Univariado. Paraná, Brasil, março de 2020 a janeiro de 2021.

É possível verificar um *cluster* alto-alto que compreende toda a 9ª RS (Foz do Iguaçú), com munícipios conglomerados que pertencem à 10ª RS (Cascavel) e 20ª RS (Toledo), bem como dois outros *clusters* alto-alto, um deles formado por cidades da 1ª RS (Paranaguá) e 2ª RS (Curitiba) e o outro formado por municípios da 7ª RS (Pato Branco) e 8ª RS (Francisco Beltrão). Um quarto *cluster* alto-alto aparece no Norte do estado, representando as cidades das regionais de Maringá e Londrina, 15ª e 17ª RS respectivamente (Figura 3).

Um *cluster* grande baixo-baixo foi formado com quase todos os municípios da 6ª RS (União da Vitória) e alguns municípios que compõem a 3ª (Ponta Grossa), 4ª (Irati), 5ª (Guarapuava), 11ª (Campo Mourão) e 12ª (Umuarama) RS, se estenderam pela região Centro-sul do estado. Além dele, outros dois *clusters* baixo-baixo apareceram na Região Norte. Um deles, formado por 14 municípios pertencentes às 18ª (Cornélio Procópio) e 19ª (Jacarezinho) RS, outro composto por seis cidades da 14ª (Paranavaí) RS e uma cidade da 15ª (Maringá) RS. Os muncípios com valores baixo-alto e alto-baixo se apresentaram em pequeno número e espalhados pelo estado (Figura 3).

Na análise bivariada, os casos confirmados de covid-19, coletados a partir do local de residência das pessoas com a doença, tiveram correlação positiva ou direta com a distribuição suavizada dos leitos de UTI, com índice de 0,085 (p ≤ 0,001). Apesar da significância estatística, a correlação entre número de leitos de UTI e casos confirmados de covid-19 foi fraca, seguindo o padrão identificado na maioria dos municípios do estado. Do total de municípios (399), em 265 (66,4%) não houve correlação significativa entre número de leitos e prevalência de covid-19. Nos 134 (33,6%) municípios restantes observou-se formação de *clusters*, com dois destaques marcantes. Um formado por municípios com forte correlação entre alto número de leitos e alta prevalência de covid-19 e outro formado por municípios com baixo número de leitos e baixa prevalência de covid-19. No *cluster* alto-alto a correlação foi 1,105 (p < 0,05), enquanto no *cluster* baixo-baixo foi 0,499 (p < 0,005).

Ao observar o *clusters* formados por municípios com alto número de leitos de UTI e alta prevalência de casos, destaca-se Curitiba e Paranaguá na macrorregional leste; Foz do Iguaçu e Toledo na macrorregional Oeste; Umuarama e Maringá na macrorregional Noroeste e Londrina e Apucarana na macrorregional norte (Figura 4). A Figura 4 identifica o *cluster* com municípios com baixo número de leitos de UTI e baixa prevalência de casos de covid-19, com destaque para Cornélio Procópio, Jacarezinho e Ivaiporã na macrorregional norte; Irati, Guarapuava e União da Vitória na macrorregional leste e Campo Mourão na macrorregional noroeste. Na macrorregional oeste observa-se um *cluster* baixo-baixo nos municípios de Guaraniaçu e Diamante do Sul na regional de Cascavel, mas agregado ao padrão da regional de Guarapuava.

**Figura 4:** Distribuição espacial da correlação entre a taxa de leitos de unidade de terapia intesiva exclusivos para covid-19 e casos confirmados da doença, segundo a análise do Índice Local de Moran (LISA) Bivariado. Paraná, Brasil, março de 2020 a janeiro de 2021.

Também se observam *outliers* formados por um grupo de municípios com alto número de leitos de UTI e baixa prevalência de casos da doença. Na macrorregional noroeste destacam o município de Colorado e Paranavaí. Ainda na macrorregional noroeste, encontram-se o *cluster* alto-baixo dos municípios de Rancho Alegre d’Oeste, Janiópolis e Mamborê. Na macrorregional leste, o *cluster* alto-baixo foi observado em Guarapuava, Turvo, Marquinho, Laranjeiras do Sul, Cantagalo e Goioxim. Geograficamente, os *clusters* alto-baixo estão na periferia dos *clusters* baixo-baixo; enquanto os *clusters* baixo-alto estão no entorno dos *clusters* alto-alto (Figura 4).

## DISCUSSÃO

Estudos que correlacionam casos confirmados de covid-19 com leitos de unidades de terapia intensiva no estado do Paraná não foram identificados na literatura científica. Conhecer essa correlação a partir de ferramentas da análise espacial permitiu identificar regiões e municípios vulneráveis no estado, compreender a relação entre o evento e os municípios que fazem vizinhança entre si e analisar o comportamento da doença no Paraná, conforme a disponibilidade de leitos de UTI exclusivos para a doença.

Estudo similar analisou a distribuição de casos de covid-19 e de leitos de terapia exclusivos para a doença no estado do Ceará^18^, demonstrou a interioriazação da doença para além da capital Fortaleza e sua região metropolitana e observou a escassez de leitos em regiões com altos índices de contaminação por covid-19.

Observa-se um crescimento exponencial e constante do número de casos confirmados e óbitos por covid-19 em todo o mundo, o Brasil aparecendo é um dos epicentros do novo coronavírus^19^. Tal crescimento pode ser justificado, basicamente, devido ao alto poder de trasmissibilidade e disseminação do vírus, falta de imunidade prévia das populações e pela inexistência de vacinação contra o novo coronavírus^20^.

Até 18 de janeiro de 2021, a prevalência de casos confirmados no Brasil era de 4.039 a cada 100 mil habitantes e o aumento no número de casos da covid-19 foi evidenciado em todas as regiões do país. Especificamente na região sul, foi constatada uma taxa de 5.470 casos confirmados da covid-19 pelo mesmo coeficiente, sendo que o Paraná é o estado da Região Sul com a menor taxa (4.339 casos confirmados – segundo resultados deste estudo), seguido do Rio Grande do Sul (4.717) e Santa Catarina (7.915)^21^ Esse ranking de classificação dos estados da Região Sul quanto ao número de casos de covid-19 vem sendo mantido desde o início da pandemia, segundo resultados apresentados em outros estudos^21^.

Embora a Região Sul e, principalmente o estado do Paraná, apresentem dados que os colocam em uma situação menos preocupante no que diz respeito aos números de casos confirmados de covid-19 em outras regiões do país, é evidente que o volume de novos casos comporta-se de forma crescente no estado^13^. Diante disso, é de suma importância o desenvolvimento de ações específicas de controle coletivo da doença, para conter a disseminação do vírus entre os municípios e as regionais de saúde do estado.

Os resultados específicos do Paraná apresentados neste estudo apontam que há importantes diferenças na distribuição dos casos confirmados de covid-19 entre os municípios e regionais de saúde do estado. As disparidades identificadas são justificadas por diversos fatores, tais como a data do início de ocorrência da doença nos municípios e regiões, densidade demográfica e populacional, distribuição etária e características das populações, condições dos serviços de saúde, controle de disseminação da doença nas comunidades, prática e fidedignidade de notificação dos casos e capacidade diagnóstica^24^.

As maiores prevalências de casos confirmados de covid-19 do Paraná foram observadas nos municípios pertencentes às regionais de saúde de Foz do Iguaçú (9ª RS), Paranaguá (1ª RS) e Francisco Beltrão (8ª RS), com valores acima da média do estado.

Destaca-se que os casos de covid-19 da grande Curitiba, localizada na macrorregional Leste, atingem diretamente Paranaguá (cidade vizinha da capital e fronteira com o estado de São Paulo)^21^. Observa-se ainda que nas 9ª e 8ª RS, pertencentes à macrorregião de saúde Oeste, ocorre a interiorização dos casos, justificada pelas características dessas regiões que são consideráveis polos comerciais do estado, ligadas à capital pelas BR-376, BR-476, BR-153 e BR-277^21^. Portanto, compreender redes e dependências sociais, econômicas, comerciais e o fluxo de viagens e transporte dentro dos limites de uma região ou estado são de suma importância para o entendimento da expansão territorial da covid-19^25^.

Já na macrorregião de saúde do Leste do Paraná, especificamente nos municípios da 6ª RS, de União da Vitória, concentraram-se as menores prevalências de casos confirmados de covid-19 (1.843 casos confirmados a cada 100 mil habitantes – menor do que o apresentado no estado). Também foi identificado nessa regional dependência espacial local significativa, apresentando baixa prevalência de casos, similares às regionais vizinhas.

Mesmo diante desses resultados, ações de prevenção e controle da doença devem ser intensificadas nessas regiões para evitar o surgimento de novos casos por meio do deslocamento das pessoas infectadas e consequente transmissão do vírus. Uma das medidas sugeridas é estimular a população na manutenção das medidas de higiene, distanciamento e isolamento social, uma vez que o vírus tem se propagado rapidamente devido à transmissão comunitária^8^. Ademais, já é evidenciado que municípios e regiões inicialmente com baixa prevalência de casos têm apresentado aumento exponencial do número de pessoas infectadas ao longo do tempo^29^.

Com relaçao ao número de leitos de UTI exclusivos para a covid-19 no Brasil, observa-se uma taxa de 9,77 leitos a cada 100 mil habitantes, sendo que a Região Sudeste apresenta a maior taxa do país (10,90 leitos). No que tange especificamente à Região Sul do país, observa-se uma prevalência de 10,14 leitos de UTI para covid-19 a cada 100 mil habitantes, sendo que o estado de Santa Catarina apresenta a maior taxa (12,82), seguido do Rio Grande do Sul (9,59) e Paraná (8,99)^14^. Evidencia-se, portanto, que o Paraná é o estado da Região Sul com a menor taxa de leitos de UTI exclusivos para a covid-19, como já demonstrado em outro estudo^28^.

Sobre as especificidades do número de leitos de UTI exclusivos para a covid-19 no estado do Paraná, os resultados deste estudo mostraram maior prevalência de leitos a cada 100 mil habitantes nos municípios pertencentes à 7ª RS – Pato Branco (52,17), 2ª RS – Curitiba (45,78) e 20ª RS – Toledo (41,99). Similarmente à prevalência de casos confirmados de covid-19, os resultados deste estudo demonstram que existe disparidade entre os municípios e regionais de saúde do Estado do Paraná quanto à distribuição do número de leitos exclusivos para a doença, sendo que em algumas RS apresentaram um maior número de leitos destinados especificamente à covid-19, como as de Curitiba, Guarapuava, Pato Branco, Campo Mourão, Maringá e Toledo.

Pela análise bivariada identifica-se um *cluster* em que a alta prevalência de casos de covid-19 se correlaciona positivamente com a alta disponibilidade de leitos de UTI exclusivos para a doença. Os achados indicam que esses municípios geralmente são sede das regionais de saúde (obedecendo à lógica do princípio de regionalização do Sistema Único de Saúde – SUS), municípios de grande e médio porte, com maior densidade demográfica e altas taxas da doença. A distribuição e a centralização dos leitos de UTI nesses municípios e regiões podem ser consideradas estratégias válidas para a homogeneidade e distribuição equitativa, porém, a distância percorrida pelo paciente para o acesso ao serviço de saúde não pode ser muito alta, devido à rápida evolução da doença^24,30,31^. Novos estudos avaliando o dimensionamento espacial dos serviços de atendimento para casos graves de covid-19 podem trazer contribuições valiosas para o conhecimento acerca desse fenômeno.

Como limitações, a coleta de dados foi realizada em um único período, sendo que os números são constantemente atualizados e, consequentemente, aumentando os casos confirmados. Quanto ao número de leitos exclusivos para a covid-19 foram considerados somente os leitos disponíveis no Paraná até o momento da coleta dos dados. E, para a comparabilidade e o aprofundamento na discussão dos achados deste estudo, não foram identificadas pesquisas sobre a distribuição geográfica da covid-19 no estado do Paraná. Contudo, este estudo pode subsidiar futuras pesquisas epidemiológicas sobre a análise espacial da covid-19 e auxiliar gestores de saúde estaduais e municipais na tomada de decisões.

Ainda assim, este estudo permitiu identificar a autocorrelação espacial direta dos casos de covid-19 no estado do Paraná, bem como a correlação positiva com os leitos exclusivos de UTI para a doença. Isso se mostra nas disparidades identificadas entre os municípios e as regionais de saúde no que diz respeito à distribuição de casos e leitos.Além disso, os *clusters* identificados pelo Índice de Moran Local, destacam áreas prioritárias de atenção no estado.
